# SIRT3 Expression Decreases with Reactive Oxygen Species Generation in Rat Cortical Neurons during Early Brain Injury Induced by Experimental Subarachnoid Hemorrhage

**DOI:** 10.1155/2016/8263926

**Published:** 2016-12-08

**Authors:** Wei Huang, Yong Huang, Ren-qiang Huang, Cheng-guang Huang, Wen-hao Wang, Jin-mao Gu, Yan Dong

**Affiliations:** ^1^Department of Neurosurgery, The 175th Hospital of PLA, Affiliated Southeast Hospital of Xiamen University, Center of Traumatic Neurosurgery in Nanjing Military Command, 269 Middle Zhanghua Road, Zhangzhou 363000, China; ^2^Department of Neurosurgery, Changzheng Hospital, Second Affiliated Hospital of Second Military Medical University, 415 Feng Yang Road, Shanghai 200003, China

## Abstract

Sirtuin3 (SIRT3) is an important protein deacetylase which predominantly presents in mitochondria and exhibits broad bioactivities including regulating energy metabolism and counteracting inflammatory effect. Since inflammatory cascade was proved to be critical for pathological damage following subarachnoid hemorrhage (SAH), we investigated the overall expression and cell-specific distribution of SIRT3 in the cerebral cortex of Sprague-Dawley rats with experimental SAH induced by internal carotid perforation. Results suggested that SIRT3 was expressed abundantly in neurons and endothelia but rarely in gliocytes in normal cerebral cortex. After experimental SAH, mRNA and protein expressions of SIRT3 decreased significantly as early as 8 hours and dropped to the minimum value at 24 h after SAH. By contrast, SOD2 expression increased slowly as early as 12 hours after experimental SAH, rose up sharply at the following 12 hours, and then was maintained at a higher level. In conclusion, attenuated SIRT3 expression in cortical neurons was associated closely with enhanced reactive oxygen species generation and cellular apoptosis, implying that SIRT3 might play an important neuroprotective role during early brain injury following SAH.

## 1. Introduction

Subarachnoid hemorrhage (SAH), especially following rupture of an aneurysm, is a devastating neurological disease associated with high morbidity and mortality [1]. Victims who survive from the initial episode frequently suffer from persistent neurological disability and poor life quality as a result of severe brain injury [2]. Although major advances have been made in surgical techniques and diagnostic radiology, the prognosis of aneurysmal SAH patients is still poor [3–5].

Recently, a large body of aneurysmal SAH literature has indicated strongly that early brain injury (EBI) might play a more pivotal role in neurological impairment and poor prognosis after SAH [6, 7]. EBI is associated with various pathophysiological processes including blood-brain barrier disruption, brain swelling, and dramatic increase of intracranial pressure, occurring within the first 72 h secondary to SAH [8]. Multiple molecular changes occur in this period, such as expression of inflammatory mediators and initiation of apoptotic cascades and oxidative stress [9, 10].

The sirtuins, as a family of highly conservative NAD^+^-dependent enzymes, have been shown to participate in transcriptional silencing and regulation of mitochondrial functions [11]. As one of the known seven members of the sirtuin family, SIRT3 is distinguished by its main localization in mitochondria, which has been proved as a key regulator in cellular protection under many pathophysiological conditions including metabolic disorders and oxidative stress [12–15]. It is suggested that SIRT3 attenuates doxorubicin-induced reactive oxygen species (ROS) output in H9c2 cardiomyocytes through deacetylating antioxidant enzymes such as superoxide dismutase 2 (SOD2) and regulating mitochondrial biogenesis such as fission, fusion, and mitophagy [16].

Although hypoxia, ischemic injury, and other types of oxidative stress are closely involved in SAH, especially at the stage of EBI [17], there are few studies concerning the function of SIRT3 as an important antioxidant mediator in cerebral cortex after SAH. Therefore, this study aimed to investigate whether SIRT3 plays a pivotal role in neuroprotection against oxidative stress induced by SAH during EBI by investigating the expression and cellular distribution of SIRT3 in cortex after SAH in a rat model.

## 2. Materials and Methods

### 2.1. Animal Preparation

All experimental procedures were approved by the Animal Care and Use Committee of Second Military Medical University and complied with the Guide for the Care and Use of Laboratory Animals by National Institutes of Health. Male Sprague-Dawley (SD) rats (280 to 330 g) were raised ordinarily and randomly divided into sham group and SAH groups (*n* = 6 for each subgroup). Rats were sacrificed at indicated time points during the following experiments.

### 2.2. Endovascular Perforation for Animal SAH Model

The endovascular perforation model was established to induce experimental SAH as described previously [18]. In brief, after anesthesia, a sharpened 4-0 monofilament nylon suture was guided into the right external carotid artery (ECA) stump and advanced into the internal carotid artery (ICA). Then the suture was advanced further to punch at the bifurcation of the anterior and middle cerebral arteries and evacuated immediately. After operation, rats were monitored ordinarily. Sham-operated rats underwent an identical procedure without perforation.

### 2.3. Neurologic Scores

Neurologic scores were evaluated by two blinded investigators, which consisted of three examinations about behavioral activity (Table 1). The indexes of appetite, activity, and neurological deficits were evaluated in the sham group and the 24 h SAH group. The grades of deficits were defined as below [19]: no neurologic deficit (score = 0); suspicious or minimum neurologic deficit (score = 1); mild neurologic deficit (score = 2-3); severe neurologic deficit (score = 4–6).

### 2.4. Brain Water Content Measurement

Rats were sacrificed 24 h after experimental SAH. Brain tissues were removed and weighed fresh (wet weight). Then the brain tissues were immediately transferred to an oven, dried at 110°C for 72 h, and weighed again (dry weight). Brain water content was determined according to the equation below: brain water content (%) = (wet weight − dry weight)/wet weight × 100%.

### 2.5. Blood-Brain Barrier (BBB) Permeability Analysis

The permeability of BBB was assessed 24 h after experimental SAH and quantified according to the method of Evans blue (EB) extravasations. Briefly, EB dye was injected through tail vein 1 h before and rinsed off just before sacrifice. All brain tissue specimens were refrigerated and centrifuged, and the absorbance of EB supernatant was detected at 610 nm by using a spectrophotometer.

### 2.6. Perfusion-Fixation and Brain Tissue Preparation

Rats were anesthetized and underwent transcardial perfusion with 0.9% ice-cold heparinized saline via the left cardiac ventricle for brain tissue preparation. For real-time PCR analysis and western blot, cortex of temporal lobe near the hematoma was harvested and preserved in liquid nitrogen. For immunohistochemistry, immunofluorescence, and TUNEL experiments, rats were perfused additionally with 4% buffered paraformaldehyde for 8 h, and the cortex of temporal lobe was embedded in paraffin and sectioned for further experiments.

### 2.7. RNA Isolation and Quantitative Real-Time PCR

RNAs were extracted from temporal cortex ordinarily. The following primers were synthesized for real-time PCR: GAPDH (5′-TTCCTACCCCCAATGTATCCG-3′ forward; 5′-CATGAGGTCCACCACCCTGTT-3′ reverse); SIRT3 (5′-CCCAATGTCGCTCACTACTTCC-3′ forward; 5′-CGTCAGCCCGTATGTCTTCC-3′ reverse); SOD2 (5′-CGTGACTTTGGTTCCTTTGAC-3′ forward; 5′-AGTGTCCCCGTTCCTTATTGA-3′ reverse).

### 2.8. Western Blot Analysis

Western blot analysis was performed as reported previously [20]. Equivalent amounts (25 *μ*g) of protein lysates were separated in each cell line. The following primary antibodies were used: anti-SIRT3 antibody (diluted 1 : 200), anti-SOD2 antibody (diluted 1 : 200), and *β*-actin (diluted 1 : 5000). The following secondary antibodies were used: HRP-conjugated anti-goat IgG (diluted 1 : 2000), HRP-conjugated anti-rabbit IgG (diluted 1 : 2000), and HRP- conjugated anti-mouse IgG (diluted 1 : 2000). All the antibodies were purchased from Santa Cruz Biotechnology.

### 2.9. Immunohistochemistry (IHC) Staining

Immunocytochemistry experiments were performed and immunoreactivity score (IRS) was interpreted by two pathologists blinded to the experiment as described previously [21]. The primary antibody was anti-SIRT3 antibody (1 : 50), and the secondary antibody was biotinylated goat anti-goat IgG (1 : 50). All the antibodies were purchased from Santa Cruz Biotechnology.

### 2.10. Immunofluorescence Staining

Immunofluorescence was carried out based on standard protocol. Tissue blocks containing the temporal cortex were ordinarily fixed, dehydrated, and cut into 6 *μ*m thick sections. The sections were first cocultured with 5% normal fetal bovine serum in PBS including 0.1% Triton X-100 for 2 h and then with anti-neuron-specific nuclear protein (NeuN) antibody (1 : 200, Millipore, USA) and anti-SIRT3 antibody (1 : 100) or anti-glial fibrillary acidic protein (GFAP) antibody (1 : 200, Millipore, USA) and anti-SIRT3 antibody (1 : 100, Santa Cruz Biotechnology Inc., USA) overnight at 4°C. Secondary antibodies included goat anti-rabbit IgG (1 : 200) and goat anti-guinea pig IgG (1 : 200, Invitrogen Life Technologies, USA). The sections were incubated with secondary antibodies and counterstained with DAPI counterstain. The fluorescence images were acquired using Nikon TE200 (Nikon Corporation Company, Tokyo, Japan) with a Spot RT digital camera.

### 2.11. TUNEL Staining

In order to evaluate cellular apoptosis in the temporal cortex, the TUNEL staining was adopted to detect the fragmentation of DNA in cell nuclei using an In Situ Cell Death Detection Kit (Roche Applied Science, Germany). Cellular apoptosis was interpreted by two pathologists blinded to the experiment. Briefly, for each slide, five high-power (400x) fields were selected randomly for quantification. Total numbers of 500 cortical cells were counted, and percentage of positive cell was evaluated for every 100 cells. Apoptosis index was confirmed by mean value of 5 percentages mentioned above. 

### 2.12. Statistical Analysis

The data were presented as means ± SEM. SPSS18.0 and GraphPad Prism 5.0 were used for statistical analysis using independent samples *t*-test and one-way analysis of variance (ANOVA) followed by least significant difference (LSD) post hoc test. Statistical significance was defined at *P* < 0.05.

## 3. Results

### 3.1. General Observation and Mortality

During the whole experimental period, all the animals showed stable weight, body temperature, mean arterial pressure, and arterial blood gas parameters (data not shown). After euthanasia, it could be detected that the blood clots were distributed near the circle of Willis, the ventral brainstem, and basilar arteries in the SAH group, but not in the sham group.

### 3.2. Evaluation of Neurologic Deficits, Brain Edema, and BBB Disruption

**Figure 1:**
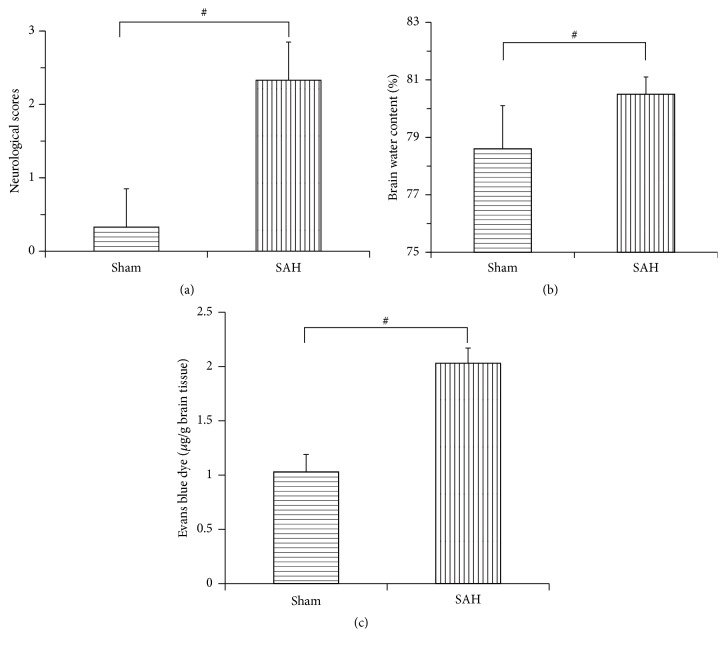
Bar graphs showing comparisons of neurologic scores, brain water content, and blood-brain barrier (BBB) permeability between sham rats and SAH rats. (a) The neurologic score reduced obviously in the SAH group as compared with the sham group at 24 h following experimental SAH. (b) The brain water content increased in cortex at 24 h after SAH. (c) Evans blue dye extravasation experiment showed a markedly increased BBB permeability in rat cortex at 24 h after SAH onset. Data were shown as mean ± SEM. ^#^ *P* < 0.01 versus sham group and *n* = 6 for each group.

To identify cerebral damage following experimental SAH, key events such as neurologic deficits, cerebral edema, and BBB disruption were assessed 24 h after experimental SAH, by comparing with conditions before SAH. As shown in Figure 1, neurological scores of rats in the SAH group increased obviously at 24 h compared with that in the sham group (2.33 ± 0.52 versus 0.33 ± 0.52, *P* < 0.01), while brain water content in the SAH group was higher than that in the sham group (80.5% ± 0.6% versus 78.6% ± 1.5%, *P* < 0.01), as well as the BBB permeability (2.03 ± 0.14 versus 1.03 ± 0.16, *P* < 0.01).

### 3.3. Reactive Oxygen Species Generation after Experimental SAH

**Figure 2:**
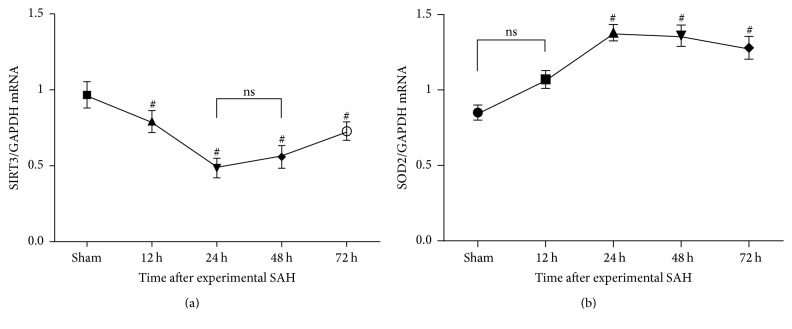
Real-time PCR analysis of SIRT3 and SOD2 mRNA levels at multiple time points after experimental SAH. (a) The mRNA level of SIRT3 decreased following SAH and was at its minimum at 24 h after experimental SAH. (b) The mRNA level of SOD2 increased slowly as early as 12 hours after experimental SAH, rose up sharply at the following 12 hours, and then was maintained at a higher level. Data were shown as mean ± SEM. Number of animals: *n* = 6 for each group and ^#^ *P* < 0.01 versus sham group, respectively; ns = not significant between two groups (*P* > 0.05).

**Figure 3:**
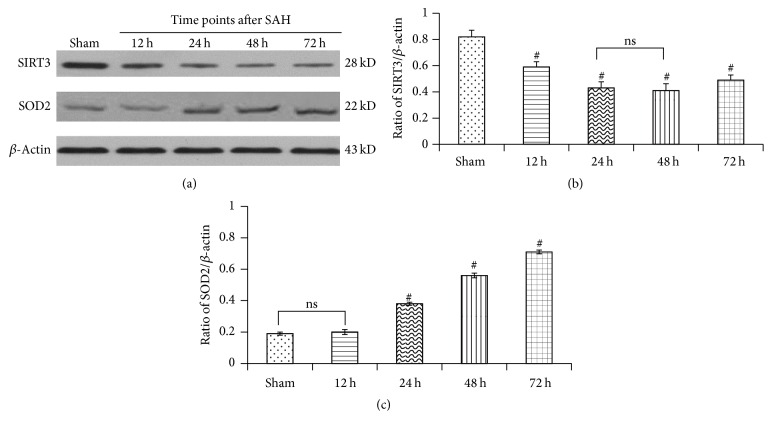
Western blot analysis of SIRT3 and SOD2 expression at multiple time points after experimental SAH. (a) Typical autoradiogram of SIRT3 and SOD2 protein expression in cortical tissue of each group. (b) Quantitative analyses. Data were shown as mean ± SEM. Number of animals: *n* = 6 for each group and ^#^ *P* < 0.01 versus sham group, respectively. ns = not significant between two groups (*P* > 0.05).

We tested the homeostatic regulation of mitochondrial reactive oxygen species (mtROS), which was associated with oxidative stress-induced injury, during the experimental aneurismal subarachnoid hemorrhage (SAH). Results suggested that both mRNA and protein expressions of SOD2 were associated inversely with the downregulation of SIRT3, which increased slowly as early as 12 hours after experimental SAH, rose up sharply at the following 12 hours, and then was maintained at a higher level (Figures 2 and 3).

### 3.4. Downregulated Expression of SIRT3 Levels after Experimental SAH

Ubiquitous SIRT3 expression was identified in sham controls. By contrast, following experimental SAH, mRNA and protein expression of SIRT3 in the temporal cortex showed slight but not statistically significant decrease at the first hours and became significant 8 and 10 hours later, respectively. Attenuation of SIRT3 expression accelerated and hit the bottom, which was nearly half as much as the sham controls, at the 24 h time points. After then, low expression of SIRT3 was maintained for about 24 hours and bounced high at the 72 h time points (Figures 2 and 3).

### 3.5. Immunohistochemical Study of SIRT3 after SAH

**Figure 4:**
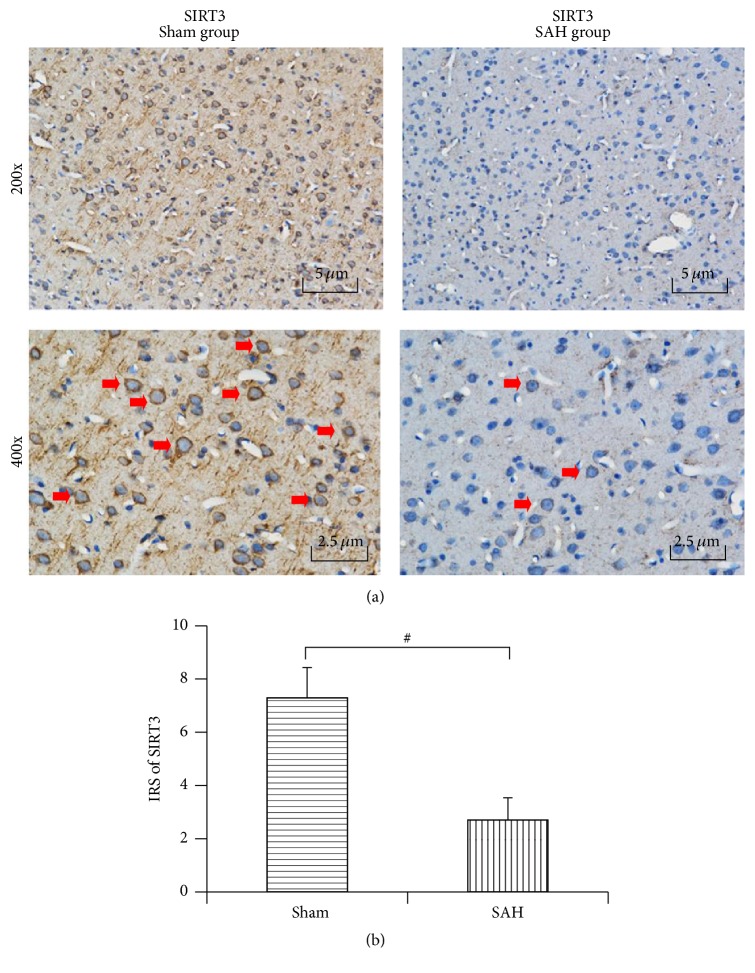
Immunohistochemistry reactivity of SIRT3 at 24 h after experimental SAH. (a) Immunohistochemistry staining results suggested that SIRT3 displayed a uniformly strong expression in the cortex of rats from the sham group (red arrows) but was significantly downregulated after experimental SAH. (b) Semiquantitative analysis revealed that immunoreactivity score of SIRT3 decreased significantly in the SAH group compared with that in the sham group. Data were shown as mean ± SEM. Number of animals: *n* = 6 for each group and ^#^ *P* < 0.01 versus sham group.

SIRT3 was expressed abundantly in the cytoplasm of neurons and endothelia but rarely in the astrocytes in the sham control. After experimental aneurysmal SAH, SIRT3 expression markedly decreased in both the neurons and endothelia, which was in accordance with the results of real-time PCR and western blotting. Semiquantitative analysis with immunoreactivity scores (IRS) showed a significant difference between the sham group and the 24 h post-SAH group (7.29 ± 1.14 versus 2.70 ± 0.84, *P* < 0.01) (Figure 4).

### 3.6. Double Immunofluorescence for the Cellular Distribution of SIRT3

**Figure 5:**
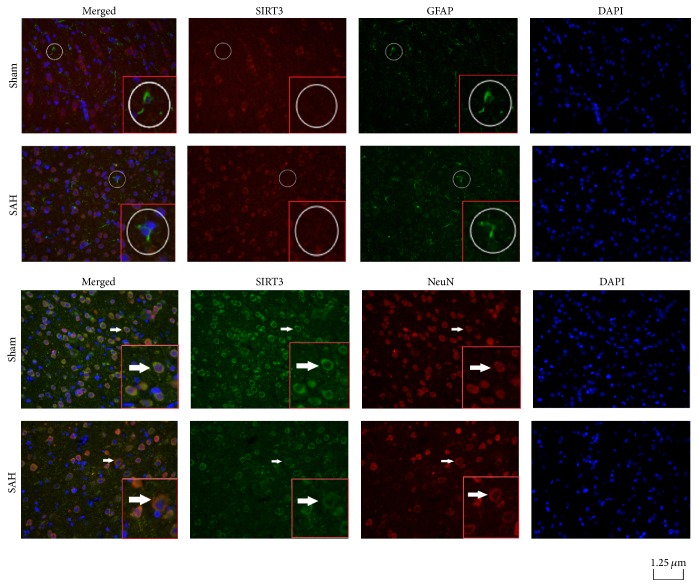
Double immunofluorescence straining for expression and cell-specific distribution of SIRT3 protein. Typical photomicrographs illustrated immunofluorescence staining of SIRT3 in cerebral cortex of rats in the sham group and the 24 h post-SAH group. NeuN, GFAP, and DAPI (blue) were used as markers for neurons, astrocytes, and nuclei, respectively. SIRT3 was seldom colocalized with GFAP in the astrocytes in all rats (white circles). By contrast, SIRT3 was abundant and colocalized with NeuN in neurons of sham groups rats (orange color in the merged photomicrographs, indicated by white arrows), which markedly decreased after experimental SAH.

In rats of the sham group, ubiquitous expression of SIRT3 was detected in NeuN-positive neurons rather than GFAP-positive astrocytes. In rats of the 24 h post-SAH group, expression of SIRT3 decreased in the cytoplasm of NeuN-positive cells but was not changed in GFAP-positive astrocytes (Figure 5).

### 3.7. Dynamic Changes of TUNEL-Positive Cells after Experimental SAH

**Figure 6:**
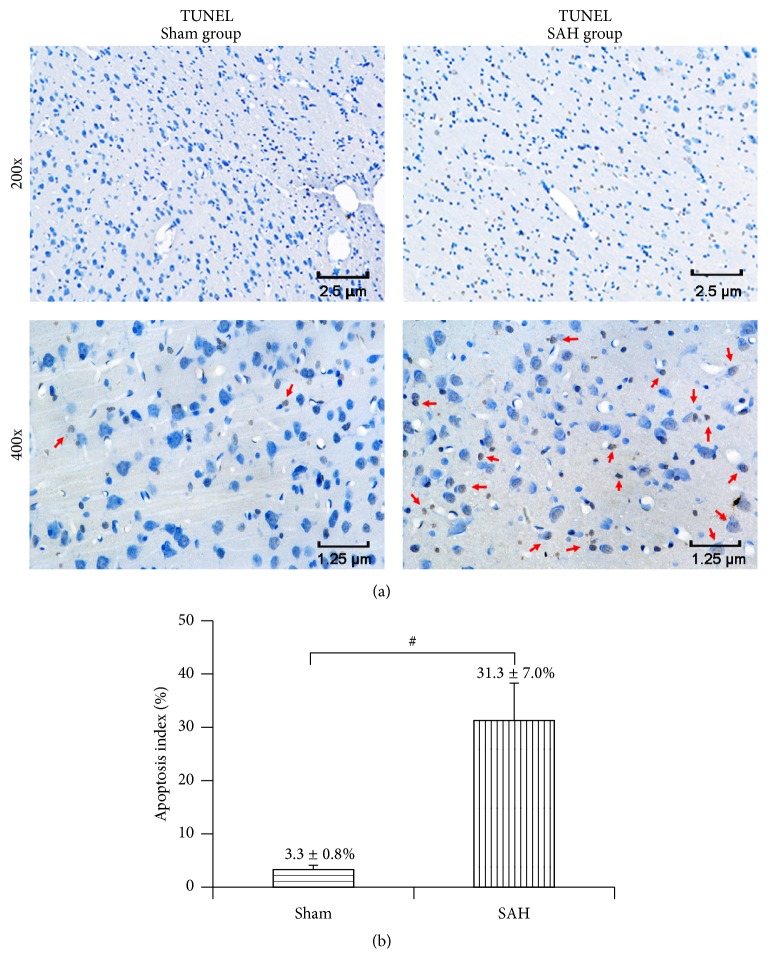
TUNEL staining for detecting apoptosis. (a) Typical photomicrographs showed that very few neurons were TUNEL-positive in the rat from the sham group. By contrast, quite many neurons (red arrows) showed moderate to strong TUNEL staining in the rat from the SAH group. (b) Quantitative analyses. Data were shown as mean ± SEM. Number of animals: *n* = 6 for each group and ^#^ *P* < 0.01 versus sham group.

In rats of the sham group, few TUNEL-positive apoptotic cells were observed in the temporal cortex, while a number of apoptotic cells were observed in the SAH group (Figure 6).

## 4. Discussion

This pilot study was designed to explore the expression and cellular distribution of SIRT3 in the cortex in an experimental SAH rat model. Results suggested that both mRNA and protein expressions of SIRT3 were downregulated since the early hours and decreased to the minimum 24 h after SAH in a time-dependent manner. IHC and immunofluorescence staining showed that SIRT3 protein was predominantly expressed in neurons, rather than in astrocytes in the lesion foci. These results suggest that SIRT3 might serve as an endogenous neuroprotective factor during the secondary early brain injury that may result in the unfavorable prognosis in SAH.

For decades, delayed vasospasm has been assumed to be the pivotal pathological mechanism for delayed neurologic deficits and poor prognosis, while the significance of EBI had not been noticed until therapies of vasospasm reversal failed to show distinct protective effects in improving long-term outcomes in clinical trials [22]. The cascade of EBI occurs promptly secondary to intracranial aneurysm rupture, which not only results in the initial clinical manifestation of SAH, but also is responsible for the delayed ischemic neurologic deficits (DIND) and unfavorable prognosis [23, 24]. Due to the significance of EBI, the hyperacute pathological changes of brain parenchyma following SAH have attracted increasing attention, including increased intracranial pressure (ICP), decreased cerebral blood flow (CBF) and cerebral perfusion pressure (CPP), breakdown of blood-brain barrier (BBB), and capillary dysfunction during the first 72 hours [25]. These pathophysiological processes could further induce global ischemic injury. Oxidative stress has been observed to play a critical role in EBI [26]. A recent* in vitro* experiment demonstrated that the upregulated protein expression of SIRT3 in PC12 cells secondary to oxygen-glucose deprivation (OGD) was an endogenous feedback response for neuroprotection [27]. Consistently, our results revealed early decrease of the expression of SIRT3 protein as well as its mRNA. Moreover, our IHC and double immunofluorescence staining results further demonstrated that SIRT3 protein was predominantly distributed in neurons but not in astrocytes. These results unanimously suggest that SIRT3 could play a critical part in the EBI cascade after experimental SAH.

Mammalian sirtuin family were initially recognized as histone deacetylases (HDAC) which are involved in a variety of physiological functions, such as stress reaction, metabolic regulation, gene silencing, and aging [28]. Previous studies have showed that SIRT3 dominantly is distributed in mitochondria and is responsible for accommodation of cellular energy stress by regulating the activity of deacetylase or ribosyltransferase, and the dynamic change of SIRT3 leads to posttranslational modification of downstream target proteins that are involved in key cellular oxidation and respiratory processes in the organelle [13]. Another study demonstrated that SIRT3 is induced to regulate adaptive oxidative stress in the circumstances of perceived energy deficiency, such as caloric restriction and fasting, and SIRT3 provides the cellular protection against oxidative damage by maintaining the homeostasis of ROS [29]. It has been recently shown that low expression of SIRT3 in cardiac tissue was induced by stress conditions [30], while, on the contrary, another study observed that the expression of SIRT3 protein was upregulated in neurons pretreated with N-methyl-D-aspartic acid (NMDA) and protected cortical neurons from excitotoxic damage via inhibition of nicotinamide adenine dinucleotide (NAD) consumption and secondary oxidative stress [31]. In the present study, we for the first time confirmed that the expression of SIRT3 was decreased in cortical neurons secondary to EBI after SAH in a time-dependent manner. Furthermore, recently a positive relationship between SIRT3 and MnSOD was observed, suggesting that SIRT3 could act as a pivotal antioxidant factor during EBI following SAH [32].

To date, considerable evidence indicates that oxidative stress is the key pathophysiological process in EBI secondary to aneurysm rupture; therefore blocking oxidative damage as early as possible could significantly improve the outcome of the patients [33]. Due to its antioxidation function, SIRT3 is considered the potential therapeutic target for EBI [26]. Although the molecular mechanisms of SIRT3 function remain inadequate and controversial, its neuroprotective role was the primary focus of discussion in recent studies. For example, an* in vitro *experiment showed that the overexpression of SIRT3 diminishes the sensitivity of PC12 neurons to both oxidative and apoptotic insults. Meanwhile, another study reported that SIRT3 protects PC12 cells against hypoxia damage through PGC-1a and MnSOD pathways by reducing ROS and maintaining ATP production [32].

In summary, this preliminary study for the first time characterized the expression pattern of SIRT3 and its cellular distribution in cerebral cortex after experimental SAH. Significant downregulation of SIRT3 was observed in the cortex during EBI. Further taking into account the previous findings from the abovementioned studies, we hypothesize that SIRT3 could provide neuroprotection effects in EBI secondary to SAH. It is necessary to comprehensively elucidate the full mechanism involved in this neuroprotection by further studies.

## Figures and Tables

**Table 1:** Behavioral scores.

Category	Behavior	Score
Appetite	Finished meal	0
	Left meal unfinished	1
	Scarcely ate	2

Activity	Walk and reach at least three corners of the cage	0
	Walk with some stimulation	1
	Almost always lying down	2

Deficits	No deficits	0
	Unstable walk	1
	Impossible to walk	2
